# Gut microbiota and functional dyspepsia: a two-sample Mendelian randomization study

**DOI:** 10.3389/fmicb.2024.1377392

**Published:** 2024-05-31

**Authors:** Yichuan Xv, Jiaxu Chen, Jiang Lin

**Affiliations:** ^1^Department of Gastroenterology, Longhua Hospital, Shanghai University of Traditional Chinese Medicine, Shanghai, China; ^2^Department of Rheumatology, Yueyang Hospital of Integrated Traditional Chinese and Western Medicine, Shanghai University of Traditional Chinese Medicine, Shanghai, China

**Keywords:** Mendelian randomization, functional dyspepsia, gut microbiota, causal effect, functional gastrointestinal disorders

## Abstract

**Background:**

Numerous studies have established that alterations in the gut microbiota (GM) constitute an embedded mechanism in functional dyspepsia (FD). However, the specific GM taxa implicated in the pathological process of FD have remained unclear.

**Methods:**

A two-sample Mendelian randomization analysis was initially conducted to examine the causal relationships between GM and FD, utilizing GWAS data from the MiBioGen Consortium (18,340 cases) and FinnGenn (8,875 cases vs. 320,387 controls). The MR study primarily employed the inverse-variance weighted (IVW) method. Sensitivity analyses were performed to test for heterogeneity and pleiotropy. Single-nucleotide polymorphisms of causal GM taxa were mapped to genes, which were subsequently assessed for causal relationships with FD employing the same methodology.

**Results:**

IVW results revealed that the genus *Clostridium innocuum group* (OR: 1.12, 95% CI: 1.02–1.24, *P* = 0.020) and genus *Ruminiclostridium 9* were positively associated with FD risk (OR: 1.27, 95% CI: 1.03–1.57, *P* = 0.028), while the genus *Lachnospiraceae FCS020 group* tended to exert a negative effect on FD risk (OR = 0.84, 95% CI: 0.73–0.98, *P* = 0.023). Among GM-related genes, a notable association was observed between RSRC1 and increased FD risk (OR = 1.13, 95% CI: 1.07–1.20, *P* < 0.001). In sensitivity analyses, no significant pleiotropy or heterogeneity of the results was found.

**Conclusions:**

This study furnished evidence for distinct effects of specific GM taxa on FD risk and hinted at a potential biological mechanism, thereby offering theoretical underpinning for future microbiotherapy of FD.

## 1 Introduction

Functional dyspepsia (FD) ranks among the most common functional gastrointestinal disorders, with a worldwide prevalence of 7–10% and is characterized by recurrent upper abdominal symptoms including premature satiety, postprandial fullness, or epigastric pain in the absence of structural abnormalities (Ford et al., 2020). Disturbed gastric accommodation, rapid or retarded gastric emptying, increased visceral sensitivity, and abnormalities in intestinal permeability have been confirmed to participate in the development of FD (Wauters et al., 2020). Although gastrointestinal dysfunction is pervasive in FD, currently available prokinetics demonstrate suboptimal efficacy in a subset of patients (Pittayanon et al., 2019). Further exploration of upstream pathologic mechanisms is urgently needed to enable more precise treatment.

A previous study has shown variations in the distribution of gut microbiota (GM) in patients with FD, and treatments based on regulating intestinal microecology have shown initial efficacy (Zhong et al., 2017). A randomized controlled trial (RCT) involving 86 patients with FD found that rifaximin, a non-absorbable systemic antibiotic, was superior to the placebo in relieving belching and postprandial fullness/bloating (Tan et al., 2017). Another recent double-blind RCT showed that a higher percentage of FD patients who consumed probiotics achieved the primary study endpoint (reduction in PDS score ≥ 0.7) compared to the placebo group (48 vs. 20%; *P* = 0.028; Wauters et al., 2021). Moreover, an observational study found that the GM profile of FD patients was completely different from that of healthy controls, even at phylum level (Fukui et al., 2020). Certain gastrointestinal microbial taxa have been proposed as likely candidate of FD symptoms, but significant heterogeneity exists among studies (Igarashi et al., 2017; Vasapolli et al., 2021), with a study even suggesting no significant differences in GM composition between FD patients and healthy individuals (Qiu et al., 2017). This may be attributed to diversity in sample collection sites and detection methods, as well as biases from dietary factors. Further elucidating the causal role of specific microbial taxa in functional dyspepsia is of paramount importance for understanding upstream pathogenic mechanisms and developing potential targets for precision therapies.

Mendelian randomization (MR) is commonly used to establish a causal relationship between exposure and outcome, which can reduce the effect of confoundings and provide justified causal sequence. In MR studies, the causal relationship is inferred utilizing single-nucleotide polymorphisms (SNPs) as instrumental variables (IVs). Because alleles are assigned randomly to the offspring and genetic information is highly conserved across disease course, the MR study can be considered as a randomized controlled trial in concept, that overcomes the limitations of traditional observation studies (Burgess et al., 2013). In our study, we comprehensively assessed the causal relationships between GM and FD based on the design of MR.

## 2 Methods

### 2.1 Study design

**Figure 1:**
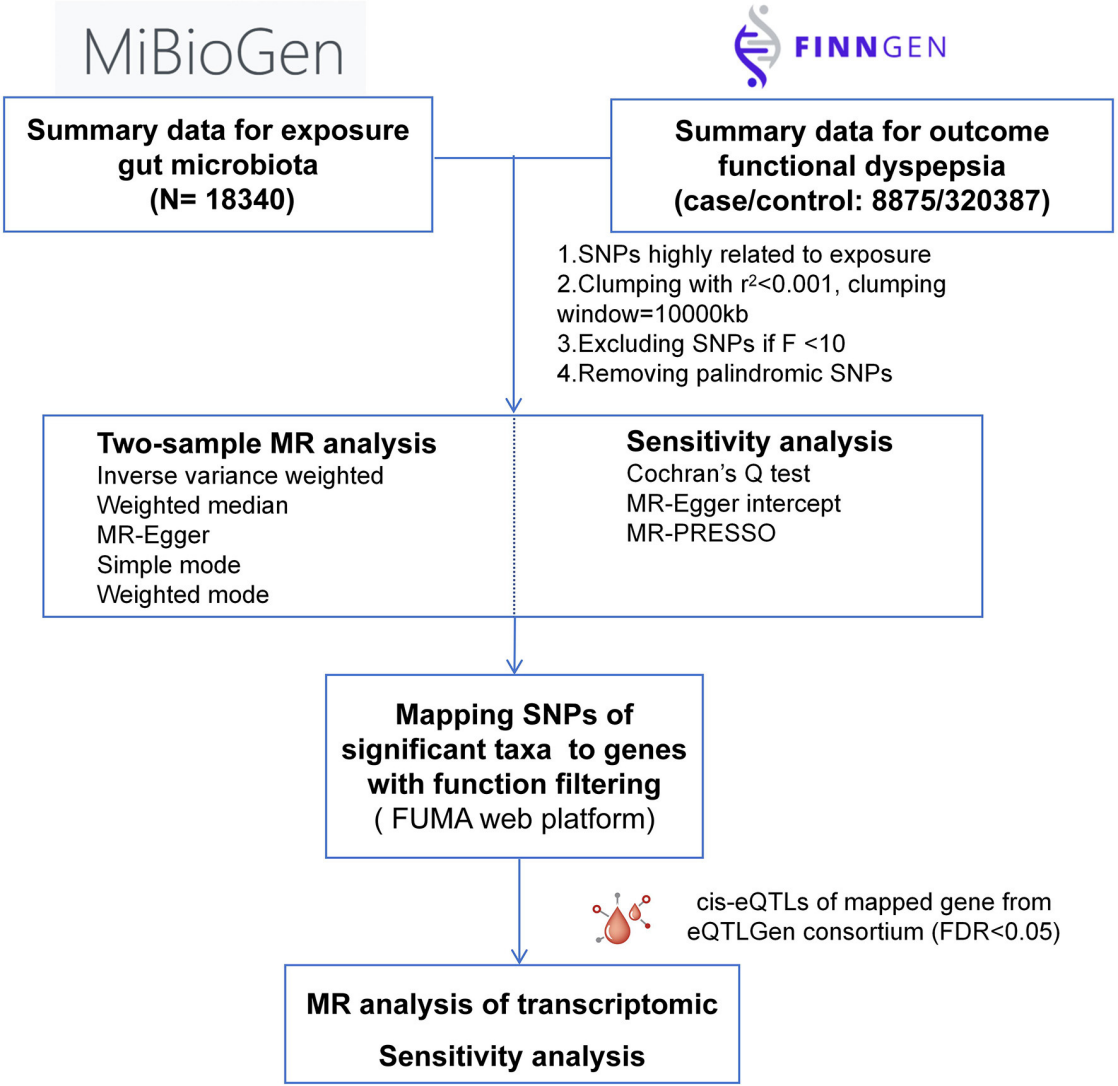
Schematic illustration of this MR study design.

We explored the causal relationship between GM and FD as well as related biological mechanisms, with summary data from MiBioGen, eQTLGene, and FinnGen consortium. The flowchart of the study design was presented in Figure 1. In MR study, three assumptions should be adhered to obtain credible findings: (i) IVs must be closely related to GM taxa (IVs); (ii) IVs were not associated with confounding factors; (iii) IVs influenced the outcome solely through exposure (Davey Smith and Hemani, 2014). This study was reported following the STROBE-MR guidelines (Skrivankova et al., 2021).

### 2.2 Data source

Summary data for the human microbiome were acquired from a genome-wide association study (GWAS) by MiBioGen Consortium. The study included 18,340 individuals across 24 cohorts in 11 countries. The description of microbial composition targets the V4, V3–V4, and V1–V2 segments of the 16S rRNA gene. The study-wide cutoffs included a minimum effective sample size of 3,000 individuals and involvement in at least three separate cohorts. A total of 211 bacterial groups were included in this study. Our study excluded 15 unknown bacterial taxa, leaving 196 bacterial taxa, which were distributed in 5 levels: phylum, class, order, family and genus (Kurilshikov et al., 2021).

FD summary data was obtained from the latest version (R9) of FinnGenn, released on May, 2023. Collectively, 8,875 patients and 320,387 controls were included in this GWAS, with FD patients defined by ICD-10 code K30, ICD-9 code 5368A, or ICD-8 code 5361 (mainly ICD-10 code; Kurki et al., 2023). All GWASs were approved by local ethical review and informed consent was obtained from every participant involved.

### 2.3 SNP selection

We first identified SNPs that were significantly associated with GM taxa (*p* < 1 × 10^−5^) and then clumped with a linkage disequilibrium (LD) coefficient of *r*^2^ = 0.001 and a window of 10,000 kb to ensure independence of all SNPs. Associations between included SNPS and other phenotypes were searched on PhenoScanner. SNPs associated with confounding factors were excluded from subsequent analyses. According to the latest review, we considered some established risk factors of FD such as gastrointestinal infection, use of antibiotics or non-steroidal anti-inflammatory drugs and emotional factors (Enck et al., 2017). In addition, we excluded SNPs associated with irritable bowel syndrome because of their high overlap in pathogenesis and frequent co-occurrence. *F*-value was calculated for each SNP to exclude weak IVs, and SNPs with *F* > 10 were considered as valid IVs.

### 2.4 Statistical analysis

Prior to the main analysis, we first aligned the effect alleles of exposure and outcome to delete SNPs with palindromic structures. According to the recommendations of the guidelines, we mainly referred to the results of the inverse variance weighted (IVW) method (Burgess et al., 2019). In this method, the wald ratio was calculated to obtain the effect of each single SNP on the outcome, and the results of all SNPs were combined by inverse variance weighted meta-analysis. If MR assumptions are not violated, this method provides the most accurate causal estimate (Burgess et al., 2013). Additional methods were applied as supplement of IVW results. The weighted median (WM) method provides more reliable results in the absence of valid instrumental variables, even if half of the information is derived from invalid instrumental variables (Bowden et al., 2016). MR-Egger method provides a intercept estimate for the test of directional pleiotropy albeit statistically less efficient (Burgess and Thompson, 2017). When disturbed by possible pleiotropy, weighted mode can identify statistically more valid causal effects with lower type I error rates (Hartwig et al., 2017). MR-PRESSO detects horizontal pleiotropy using global test and can eliminate it by removing significant outliers (Verbanck et al., 2018). Simple model method is less biased than other methods, but less precise (Hartwig et al., 2017). MR results were expressed with odds ratio (OR) and 95% confidence interval (CI).

We carried out additional sensitivity tests to assess the reliability of the results. Cochran's *Q*-test and MR-Egger intercept test were used to evaluate heterogeneity and pleiotropy, respectively (Hemani et al., 2017). Leave-one-out analysis was conducted to rule out probable significant effect of a single SNP (Luo et al., 2022). The Bonferroni correction was applied to control Class I errors in multiple tests. In our study, results with *P* < 2.6 × 10^−4^ (0.05/196) were considered as significant evidence, and results with 2.6 × 10^−4^<*P* < 0.05 were suggestive significant. All MR analyses were conducted using TwoSampleMR (version 0.5.6) package in R.

### 2.5 Mapping SNPs to genes

To gain deeper insight into how GM influences FD, SNPs of each significant taxa identified in preceding MR analyses were mapped to genes employing the SNP2GENE tool in FUMA (a platform integrating multiple databases and enabling annotation and interpretation of GWAS results; Watanabe et al., 2017). To understand protein-level gene interactions, PPI networks were generated for these GM-related genes using Metascape and were displayed with Cytoscape.

### 2.6 MR analysis of GM-related genes

We further assessed the causal effects of these genes on FD to uncover the underlying mechanism of GM influence on FD. Cis-expression quantitative trait loci (cis-eQTLs) of these gene were acquired from eQTLGen consortium. The summary data of eQTLGen consortium, enrolling totally 31,684 blood samples, contains cis-eQTLs for 16,987 genes, mostly obtained from participants of European descent (Võsa et al., 2021). We obtained valid IVs for 30 genes, with FDR < 0.05. These eQTLs were clumped by applying a relatively loose LD threshold of *r*^2^ < 0.1, which was also applied in previous MR studies (Cao et al., 2023). The statistic method for MR analysis was the same as above, and the same Bonferroni method was employed to correct for multiple tests.

## 3 Results

### 3.1 Associations of GM with FD

**Figure 2:**
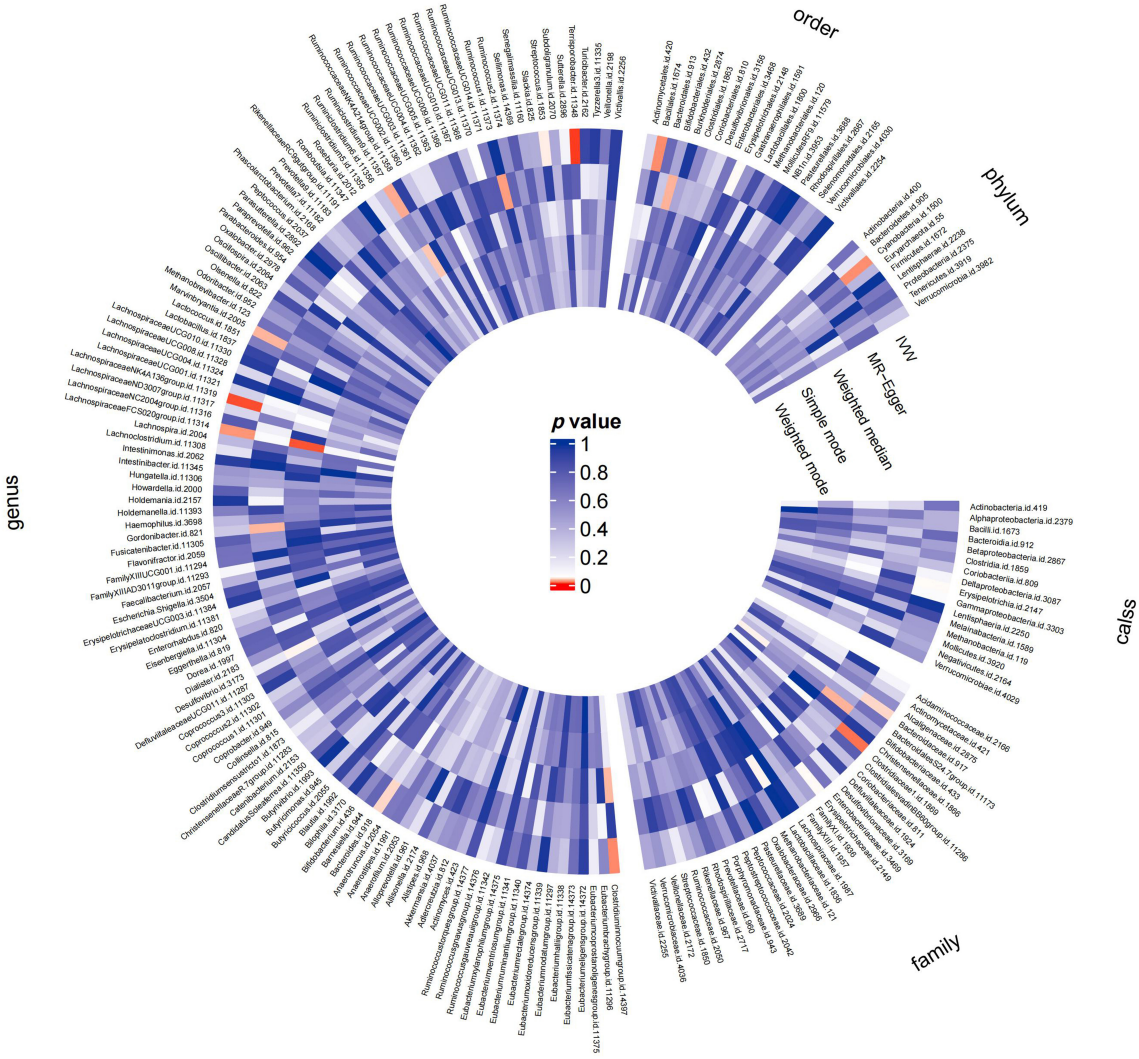
MR results of the effects of all GM taxa on FD. Gut microbiota was classified in five levels, namely order, phylum, class, family, and genus. The shade of the color reflected the size of the *p*-value within the circle.

**Figure 3:**
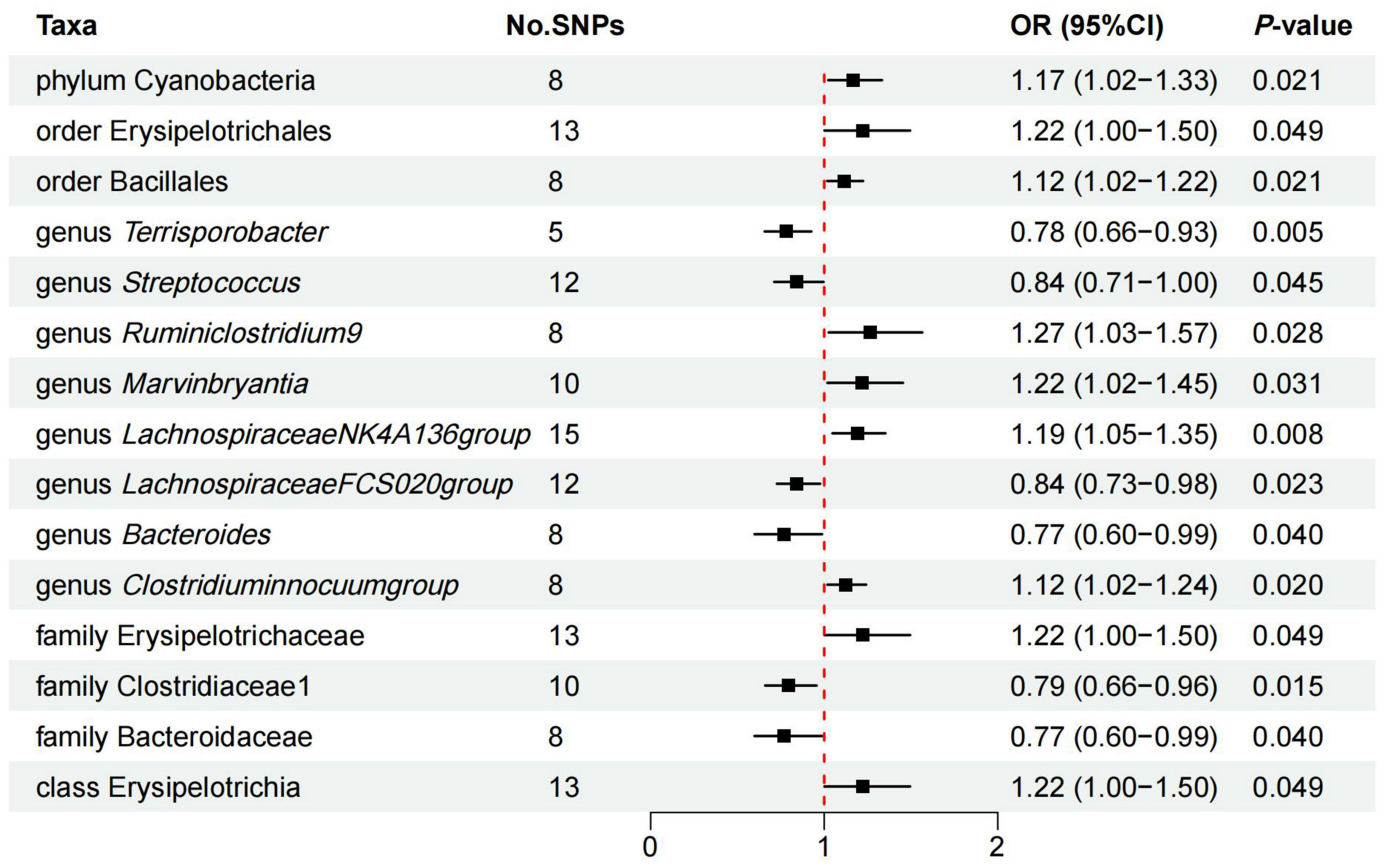
IVW estimates of the effects of significant GM taxa on FD.

**Figure 4:**
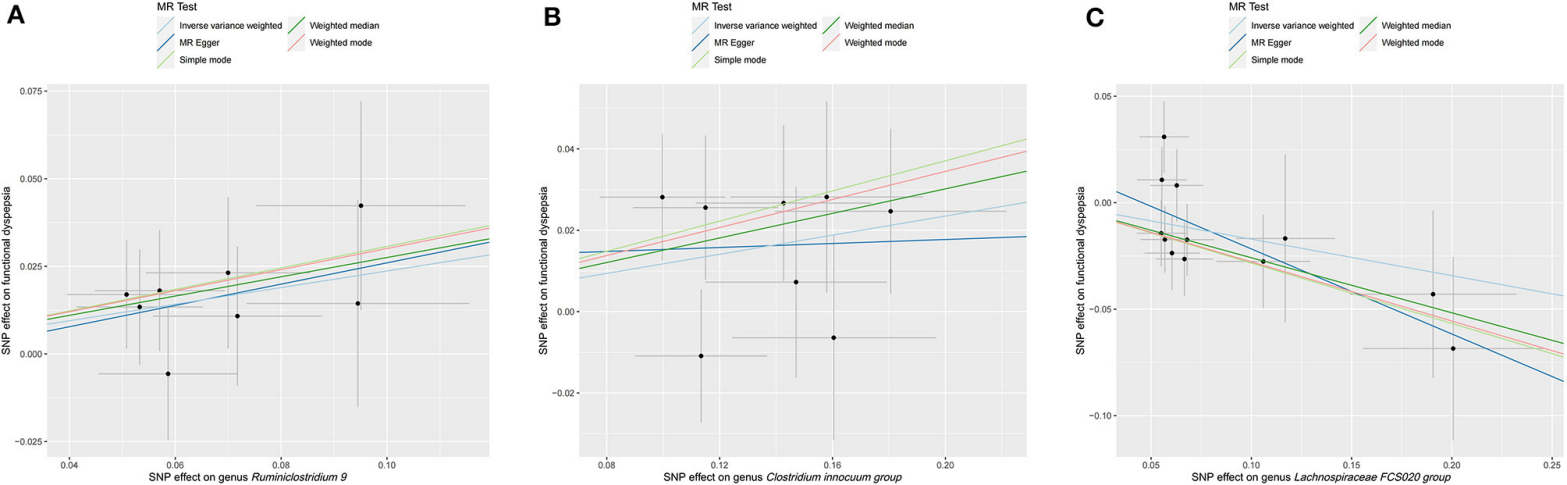
Scatter plot of MR results for five approaches to three significant GM taxa. **(A)** Scatter plot of MR results for genus *Ruminiclostridium 9*. **(B)** Scatter plot of MR results for genus *Clostridium innocuum group*. **(C)** Scatter plot of MR results for genus *Lachnospiraceae FCS020 group*.

Preliminary MR results for associations between 196 GM taxa and FD were presented in Figure 2. Briefly, we observed suggestive evidence for 3 GM taxa causally associated with FD by the cross validation of IVW and WM method (Table 1 and Figure 3). Genus *Clostridium innocuum group* was found to be positively associated with FD risk with IVW method (OR: 1.12, 95% CI: 1.02–1.24, *P* = 0.020), which was further validated by WM method (OR: 1.16, 95% CI: 1.01–1.33, *P* = 0.032). The causal evaluation from the MR-Egger analysis also supported consistent correlation (OR = 1.02, 95% CI: 0.62–1.69, *P* = 0.926; Table 1 and Figure 4B). In terms of genus *Ruminiclostridium 9*, it was also positively correlated with the risk of FD with IVW method (OR: 1.27, 95% CI: 1.03–1.57, *P* = 0.028). WM method (OR = 1.32, 95% CI 1.02–1.70, *P* = 0.036) and MR-Egger method (OR = 1.36, 95% CI: 0.49–3.73, *P* = 0.578) also exhibited a consistent trend (Table 1 and Figure 4A). In contrast, genus *Lachnospiraceae FCS020 group* was negatively associated with FD risk using IVW method (OR = 0.84, 95% CI: 0.73–0.98, *P* = 0.023). WM analysis produced similar and significant estimates (OR =0.77, 95% CI: 0.64–0.94, *P* = 0.009). The estimate of MR-Egger was similar to the IVW result but with a wider CI (OR = 0.67, 95% CI: 0.46–0.98, *P* = 0.067; Table 1 and Figure 4C). It appeared unlikely that pleiotropy or heterogeneity would bias the casual estimates, according to the results of sensitivity analyses (Table 1). Leave-one-out analyses revealed that the causative estimates of GM and FD were not attributable to a single SNP (Supplementary Figure 1).

**Table 1:** MR results of the causal associations of genus *Clostridium innocuum group*, genus *Lachnospiraceae FCS020 group*, and genus *Ruminiclostridium 9* with FD.

**Taxa**	**Method**	**No. SNP**	**OR (95% CI)**	***P*-value**	**Heterogenenity**		**Pleiotropy**		**MR-PRESSO global test**
					***Q*** **-value**	***p***	**Egger intercept**	***p***	***p***
Genus *Clostridium innocuum group*	IVW	8	1.12 (1.02–1.24)	0.020	5.43	0.61	/	/	
Genus *Clostridium innocuum group*	MR Egger	8	1.02 (0.62–1.69)	0.926	5.29	0.51	0.01	0.72	0.67
Genus *Clostridium innocuum group*	Weighted median	8	1.16 (1.01–1.33)	0.032	/	/	/	/	
Genus *Lachnospiraceae FCS020 group*	IVW	12	0.84 (0.73–0.98)	0.023	11.58	0.40	/	/	
Genus *Lachnospiraceae FCS020 group*	MR Egger	12	0.67 (0.46–0.98)	0.067	9.97	0.44	0.02	0.23	0.46
Genus *Lachnospiraceae FCS020 group*	Weighted median	12	0.77 (0.64–0.94)	0.009	/	/	/	/	
Genus *Ruminiclostridium 9*	IVW	8	1.27 (1.03–1.57)	0.028	1.95	0.96	/	/	
Genus *Ruminiclostridium 9*	MR Egger	8	1.36 (0.49–3.73)	0.578	1.94	0.93	−0.01	0.90	0.97
Genus *Ruminiclostridium 9*	Weighted median	8	1.32 (1.02–1.70)	0.036	/	/	/	/	

MR, Mendelian randomization; CI, confidence interval; OR, odds ratio; IVW, inverse variance-weighted.

Additionally, we identified latent causal relationships between other 12 taxa and FD with IVW method. Among them, seven taxa were found to have potential positive causal effect on FD, namely order phylum Cyanobacteria (OR = 1.17, 95% CI: 1.02–1.33, *P* = 0.021), order Erysipelotrichales (OR = 1.22, 95% CI: 1.00–1.50, *P* = 0.049), order Bacillales (OR = 1.12, 95% CI: 1.02–1.22, *P* = 0.021), genus *Marvinbryantia* (OR = 1.22, 95% CI: 1.05–1.45, *P* = 0.042), genus *Lachnospiraceae NK4A136 group* (OR = 1.19, 95% CI: 1.05–1.35, *P* = 0.008), family Erysipelotrichaceae (OR = 1.22, 95% CI: 1.00–1.50, *P* = 0.049), and class Erysipelotrichia (OR = 1.22, 95% CI: 1.00–1.50, *P* = 0.049; Figure 3). On the contrary, five taxa tended to causally reduce risk of FD, specifically containing genus *Streptococcus* (OR = 0.84, 95% CI: 0.71–1.00, *P* = 0.045), genus *Terrisporobacter* (OR = 0.78, 95% CI: 0.66–0.93, *P* = 0.005), genus *Bacteroides* (OR = 0.77, 95% CI: 0.60–0.99, *P* = 0.040), family Bacteroidaceae (OR = 0.77, 95% CI: 0.60–0.99, *P* = 0.040), and family Clostridiaceae (OR = 0.79, 95% CI: 0.66–0.96, *P* = 0.015; Figure 3). However, results from WM method did not support these causal associations.

### 3.2 Mapped genes of causal SNPs

**Figure 5:**
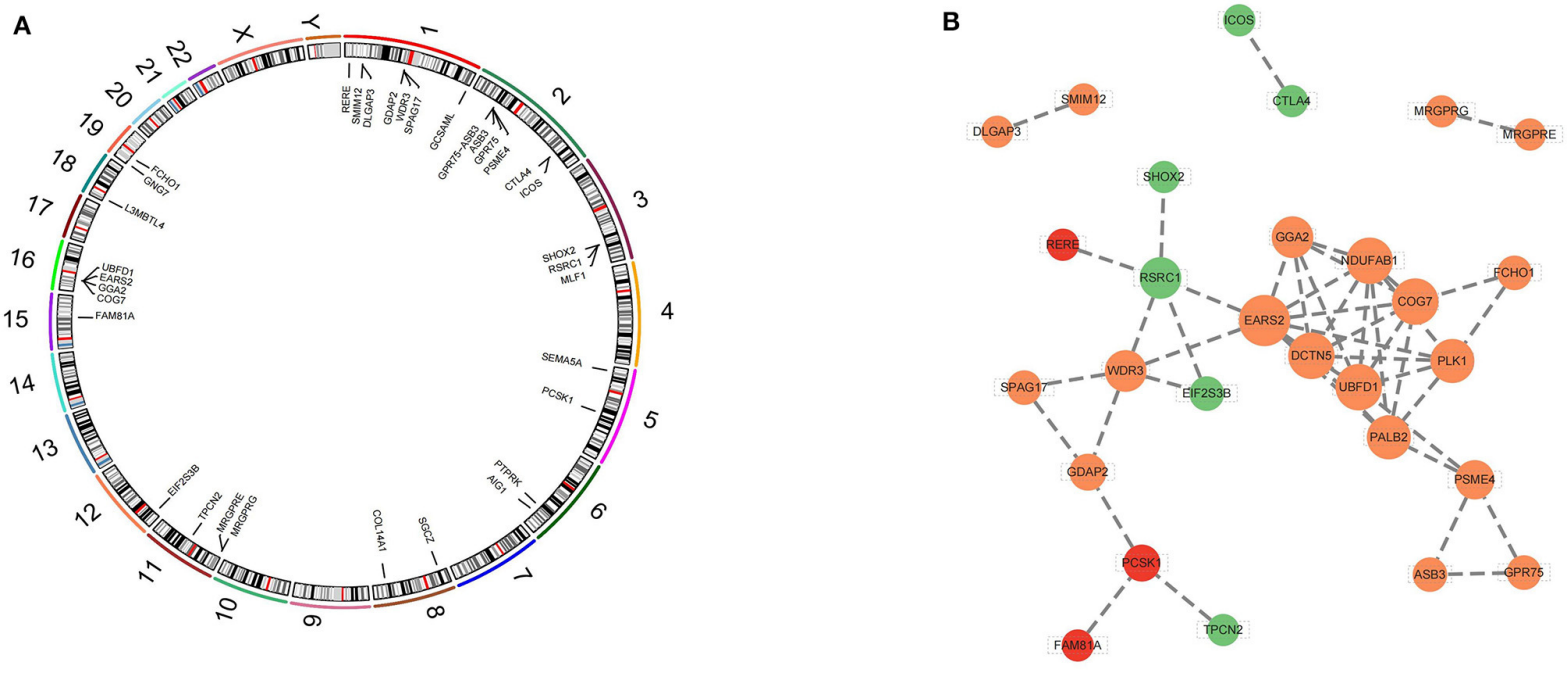
Mapped genes of GM related SNPs. **(A)** The chromosome location of mapped genes. **(B)** PPI networks from mapped genes. Red circles represented genes associated with genus *Clostridium innocuum group*, green circles represented genes associated with genus *Ruminiclostridium 9*, orange circles represented genes associated with genus *Lachnospiraceae FCS020 group*.

After the validation of additional MR methods and sensitivity analysis, three taxa, namely genus *Clostridium innocuum group*, genus *Ruminiclostridium 9* and genus *Lachnospiraceae FCS020 group* were demonstrated to have causal effect on FD. To better comprehend the biological mechanism of these findings, we perform positional mapping of the extracted SNPs of significant GM taxa with FUMA GWAS tool. IVs of significant GM taxa were shown in Supplementary Table 2. Genes mapped by SNPs located in the protein coding region were selected and enrolled in further analysis (Supplementary Table 3). The chromosome location of mapped genes were shown in Figure 5A. Protein-protein interaction networks from mapped genes were generated using Metascape, and were subsequently visualized in Cytoscape to predict the interactions of these genes, as shown in Figure 5B.

### 3.3 MR analysis based on mapped genes

**Figure 6:**
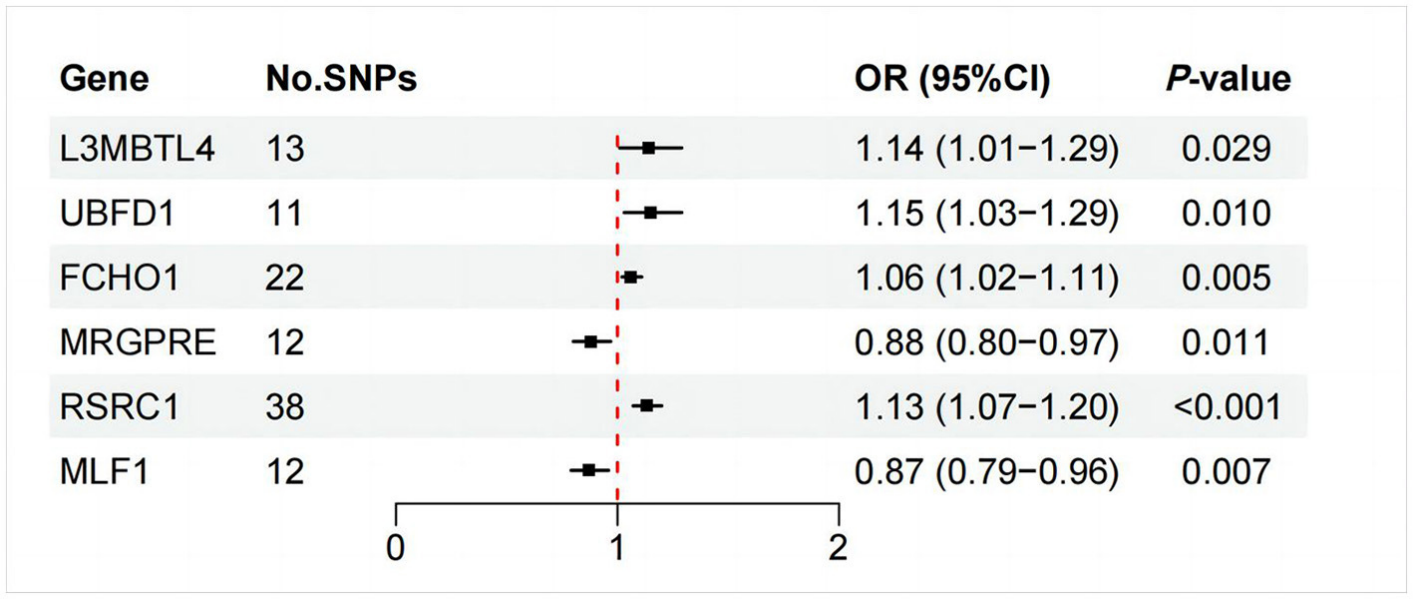
IVW estimates of the effects of significant GM mapped genes on FD.

**Figure 7:**
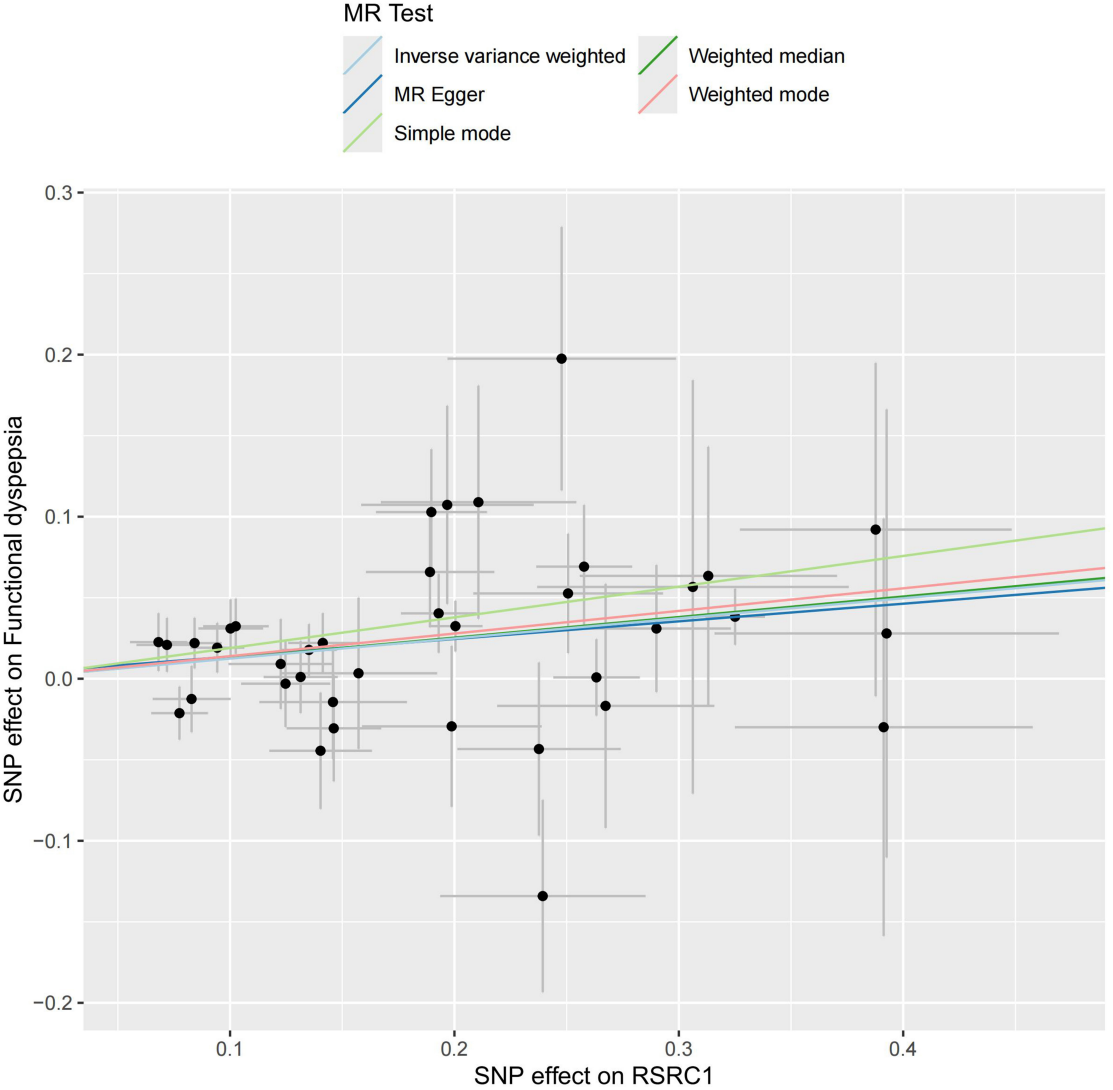
Scatter plot of MR results for five approaches to RSRC1.

As illustrated in Figure 6, only RSRC1, associated with genus *Ruminiclostridium 9*, was found to significantly increase FD risk after Bonferroni correction (OR = 1.13, 95% CI: 1.07–1.20, *P* < 0.001). All five MR approaches generated consistent estimates with the same effect direction (Table 2 and Figure 7). According to the results of intercept test and Cochrane's *Q*-test, there was implausible to be horizontal pleiotropy or heterogeneity among individual eQTLs. The expression of other five genes was suggestively associated with the risk of FD (0.0017 < *P* < 0.05).

**Table 2:** MR results of the relationship between RSRC1 and FD.

**Gene**	**Method**	**No. SNP**	**OR (95% CI)**	***P*-value**	**Cochrane's** ***Q*****-test**		**Intercept test**	
					***Q*** **-value**	***p***	**Egger intercept**	***p***
RSRC1	MR Egger	38	1.11 (0.99–1.26)	0.091	45.03	0.14	< 0.01	0.78
RSRC1	Weighted median	38	1.14 (1.05–1.23)	0.001	/	/	/	/
RSRC1	IVW	38	1.13 (1.07–1.20)	< 0.001	45.13	0.17	/	/
RSRC1	Simple mode	38	1.21 (1.06–1.38)	0.007	/	/	/	/
RSRC1	Weighted mode	38	1.15 (1.06–1.25)	0.002	/	/	/	/

MR, Mendelian randomization; CI, confidence interval; OR, odds ratio; IVW, inverse variance-weighted.

## 4 Discussion

This was the first study to uncover that partial GM taxa could induce or prevent FD using MR methods. With cross-validation of IVW and WM methods, preliminary MR results provided evidence that genus *Lachnospiraceae FCS020 group* was correlated with a lower probability of developing FD, while genus *Clostridium innocuum group* and genus *Ruminiclostridium 9* may be the risk factors of FD.

Luminal dysbiosis is widely recognized to be involved in the pathogenesis of FD. Changes in gut microbiota can affect the activity of digestive enzymes and subsequently impact the host's digestive function (Zhou et al., 2024).

Duodenal mucosal bacterial load increases accompanied with decreased diversity in FD and is negatively correlated with the quality of life. Researchers have found that the abundance of *Prevotella, Veillonella*, and *Actinomyces* was significantly decreased in patients with FD, but the abundance of *Streptococcus* exhibited an inverse trend (Zhong et al., 2017). Another study showed that the excessive abundance of genus *Clostridium* and *Prevotella* was observed in FD, partially in line with our results (Nakae et al., 2016). However, microbiome shifts showed in aforementioned studies can also be attributed to pharmacotherapeutic interventions during study period [e.g., proton pump inhibitors (Perry et al., 2020) or antidepressants (McGovern et al., 2019)] and may suggest biased connections, so that these alterations should be interpreted cautiously when inferring the etiology of FD.

In our study, genus *Lachnospiraceae FCS020 group* was identified as a protect factor of FD, while other two taxa as risk factors, namely genus *Clostridium innocuum group* and genus *Ruminiclostridium 9*. These GM taxa shape the severity of FD possibly through key mechanisms associated with short-chain fatty acids (SCFAs), mucosal inflammation, and gut-brain axis. In early studies, inhibited gastric tone and accelerated intestinal motility were observed after intracolonic infusions of SCFAs (Ropert et al., 1996; Dass et al., 2007). Moreover, the rapid duodenal SCFA absorption plays a vital role in the inhibition of luminal bacterial colonization and is associated with the development of FD caused by the bacterial overgrowth (Kaji et al., 2015). The Lachnospiraceae is a family of anaerobic bacteria in the Clostridiales order within the Firmicutes phylum. Lachnospiraceae is a major producer of short-chain fatty acids, effectively regulating gut homeostasis and improving duodenal permeability. A clinical study found a significant correlation between increased abundance of Lachnospiraceae and alleviation of colonic inflammation and improved quality of life in patients with ulcerative colitis (Facchin et al., 2020). However, it should be noted that Lachnospiraceae could increase the concentration of secondary bile acids by promoting primary bile acids to convert to secondary bile acids. The higher ratio of secondary/primary bile acids has been reported to be positively correlated with duodenal permeability in FD patients, suggesting that its potential pathogenic effect on FD (Byndloss et al., 2017; Beeckmans et al., 2020). Hence, the role of *Lachnospiraceae FCS020 group* in the pathophysiology of FD should be further investigated in the future.

Mild inflammation of the duodenal mucosa, presenting as increased eosinophils and mast cells, has been observed in FD patients. These cells release the mediators to activate sensitive neurons and breach the epithelial barrier, leading to visceral hypersensitivity and abnormal gastric motility (Vanuytsel et al., 2023). An elevation in small bowel homing T lymphocytes is also correlated with symptom severity and retarded gastric emptying in FD patients (Liebregts et al., 2007). *Clostridium innocuum group* can compromise the epithelial layer lining the gut by strengthening oxidative damage in intestinal epithelial cell (Adesso et al., 2019) and negatively affect tight junction proteins, which in turn lead to a cytokine-rich mucosal environment perpetuating inflammatory response (Huang et al., 2022). In contrast, Lachnospiraceae are enriched in proximity to the mucosa, thereby situating them to positively influence the host epithelium and mucosal immune system (Riva et al., 2019). Specifically, the immunomodulatory molecule polysaccharide acts in unison with butyrate produced by Lachnospiraceae to promote regulatory T cell differentiation, which was crucial for preventing excessive immune inflammation (Riva et al., 2019).

Peripheral etiologies aside, the role of microbiota in FD may be associated with central factors. The microbiota-mediated gut-brain axis has been extensively discussed (Rupp and Stengel, 2022). Positron-emission tomography showed that FD patients exhibited lower Sensorimotor Network and salience network activation threshold and failure of pain-related perigenual anterior cingulate cortex activation, which overlaps with central mechanisms associated with anxiety and depression (Van Oudenhove et al., 2010). Neuroinflammation driven by GM has a profound effect on brain structure and function, and these changes lead to the expression of definite psychosomatic disorders such as fatigue, hyperalgesia, and abnormal pain (Felger, 2018). GM produce a host of neuroactive metabolites that reach the brain via circulation (Valles-Colomer et al., 2019), and modulate central nervous activities. On the other hand, impaired gut barrier enables the entry of pathogenic bacteria and their products into circulatory system, and aggravate inflammation of neurons (Dinan and Cryan, 2017). Genus *Ruminiclostridium 9* and *Clostridium innocuum group* were elucidated to be positively associated with the risk of FD, whose mechanism may involve brain-gut interaction. A prospective study found that neuropsychiatric symptoms and fatigue after the recovery of COVID-19 infection were correlated with the enrichment of *Clostridium innocuum* in lumen (Liu et al., 2022). In line with these findings, another study identified the causal relationships between *Clostridium innocuum* and neuroticism (Ni et al., 2021). A longitudinal study showed that more abundance with *Ruminiclostridium 9* was related to psychoneurological symptoms patients with head and neck cancers (Bai et al., 2020). Excessive *Ruminiclostridium 9* is related to synaptic dysfunction characterized by neuroinflammation. Correcting the increase of *Ruminiclostridium 9* is beneficial to the amelioration of neuroinflammation, along with the suppression of NF-κB pathway and the upregulation of CREB/BDNF/TrkB pathway (Lan et al., 2023). Therefore, these GM taxa may affect the function of the relevant brain regions, thus resulting in stomach upset and psychological disorders.

As for MR results of GM mapped genes, the expression of RSRC1 was in a strikingly negative relation to FD. RSRC1 is widely expressed throughout the human brain according to data from Human Brain Atlas (http://www.human.brain-map.org/). The expression of RSRC1, which is also a hub gene in the PPI network of depression-related genes, is significantly up-regulated in depression, demonstrating its critical role of in the pathological process of depression. Related genes downstream of RSRC1 are also involved in other psychiatric manifestations, such as temper tantrums (Perez et al., 2018). In our study, we identified a close interaction between RSRC1 expression and genus *Ruminiclostridium 9* based on the gene mapping of GM-related SNPs, which was not reported before. The expression of certain genes can significantly modulate the composition of intestinal microbiota. It has been found that compared to normal controls, the deficiency of the TLR4 can lead to a significant increase in the abundance of genus *Ruminiclostridium 9* (Xiao et al., 2019). Hence, it is plausible to infer that up-regulated RSRC1 expression may activate the affective center and related cortical circuit, and impair the descending modulatory system where pain transmission is modulated, thereby triggering or amplifying perceptions of stomach displeasure even under physiological stimuli. The results of the other genes did not reach a significant threshold after Bonferroni correction, still awaiting more studies in future.

This study has several advantages. Building upon the MR design, we employed two approaches to cross-validate the causal impact of intestinal flora on FD. Through this methodology, we were able to provide potential candidates for microbial-related targets. Future randomized controlled trials can be designed to further explore the efficacy of microbial therapy targeting these significant GM taxa in FD. Moreover, we have elucidated the pivotal role of RSRC1 in establishing a causal link between the genus *Ruminiclostridium 9* and FD, thereby offering novel insights into the exploration of the molecular mechanisms governing microbial modulation of gastrointestinal motility. Importantly, FD encompasses two distinct subtypes, namely postprandial distress syndrome and epigastric pain syndrome, each associated with distinct microbial profiles as reported previously (Tziatzios et al., 2021). Subsequent investigations could delve into this interaction within both subtypes, thereby advancing the comprehension of the pathological mechanisms underlying the microbiome's involvement in FD.

There are also some limitations in this study. First of all, the summary data used in our study only involved the European, but given the global prevalence of FD, future MR studies based on other populations should be conducted to improve the generalizability of the conclusion. Second, the variability of the MiBioGen meta-analysis is relatively high. Future GWAS studies should employ more advanced methods such as shotgun metagenomic sequencing analysis to generate high-resolution GM data for more accurate results of future MR studies. Finally, after Bonferroni correction, our results of GM effect on FD were not significant. However, due to biological plausibility, applying a strict correction method may be too cautious to overlook possible pathogenic strains.

## 5 Conclusions

Our study thoroughly analyzed the causal effect of GM on FD risk. We found that the altered abundance of specific bacteria, namely genus *Lachnospiraceae FCS020 group*, genus *Clostridium innocuum group* and genus *Ruminiclostridium 9*, may significantly contribute to the etiology of FD. Further analysis found that RSRC1 gene may be involved in the mechanism of the causal relationship. These findings hold promise for the future microbial therapy of FD.

## Data availability statement

The original contributions presented in the study are included in the article/Supplementary material, further inquiries can be directed to the corresponding author.

## Ethics statement

Ethical approval was not required for the study involving humans in accordance with the local legislation and institutional requirements. Written informed consent to participate in this study was not required from the participants or the participants' legal guardians/next of kin in accordance with the national legislation and the institutional requirements.

## Author contributions

YX: Conceptualization, Data curation, Methodology, Software, Writing – original draft, Writing – review & editing. JC: Formal analysis, Methodology, Software, Validation, Visualization, Writing – review & editing. JL: Conceptualization, Formal analysis, Project administration, Resources, Supervision, Validation, Writing – review & editing.

## Supplementary material

Supplementary Figure 1 Leave-one-out analysis results of **(A)** genus *Clostridium innocuum group*, **(B)** genus *Lachnospiraceae FCS020 group*, and **(C)** *genus Ruminiclostridium 9*.

Supplementary Table 1 MR results of all GM taxa.

Supplementary Table 2 Information of instrument variables of significant GM taxa.

Supplementary Table 3 SNPs mapped genes using FUMA SNP2GENE tool.
