# Supplementary figures and images for: Gut microbiota and functional dyspepsia: a two-sample Mendelian randomization study
Source: Front Microbiol. 2024 May 31;15:1377392. doi: 10.3389/fmicb.2024.1377392 (PMC11176457; doi:10.3389/fmicb.2024.1377392)

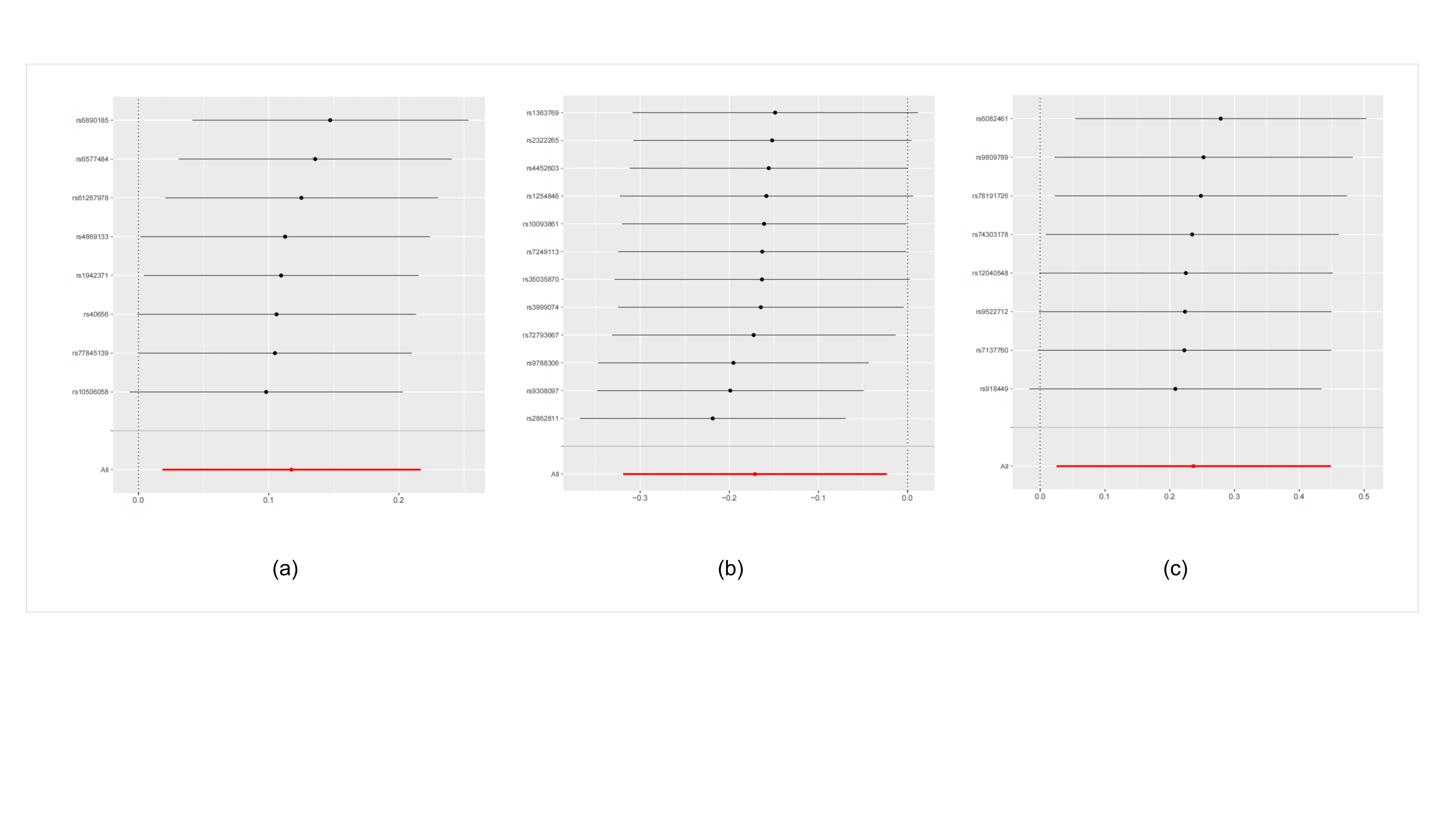

Supplement: Supplementary Figure 1 — Leave-one-out analysis results of (A) genus Clostridium innocuum group, (B) genus Lachnospiraceae FCS020 group, and (C) genus Ruminiclostridium 9. [file Image_1.JPEG]
